# Supplementary material for: Splice-Junction-Based Mapping of Alternative Isoforms in the Human Proteome
Source: Cell Rep. Author manuscript; Available in PMC 2020 Jan 15. (PMC6961840; doi:10.1016/j.celrep.2019.11.026)
Supplement: 2 [file NIHMS1546469-supplement-2.zip › DF1/PXD009737/TestisGluC/TestisGluC_13_ART3_YFQPIYVYNPGEKNWKLE.pdf]

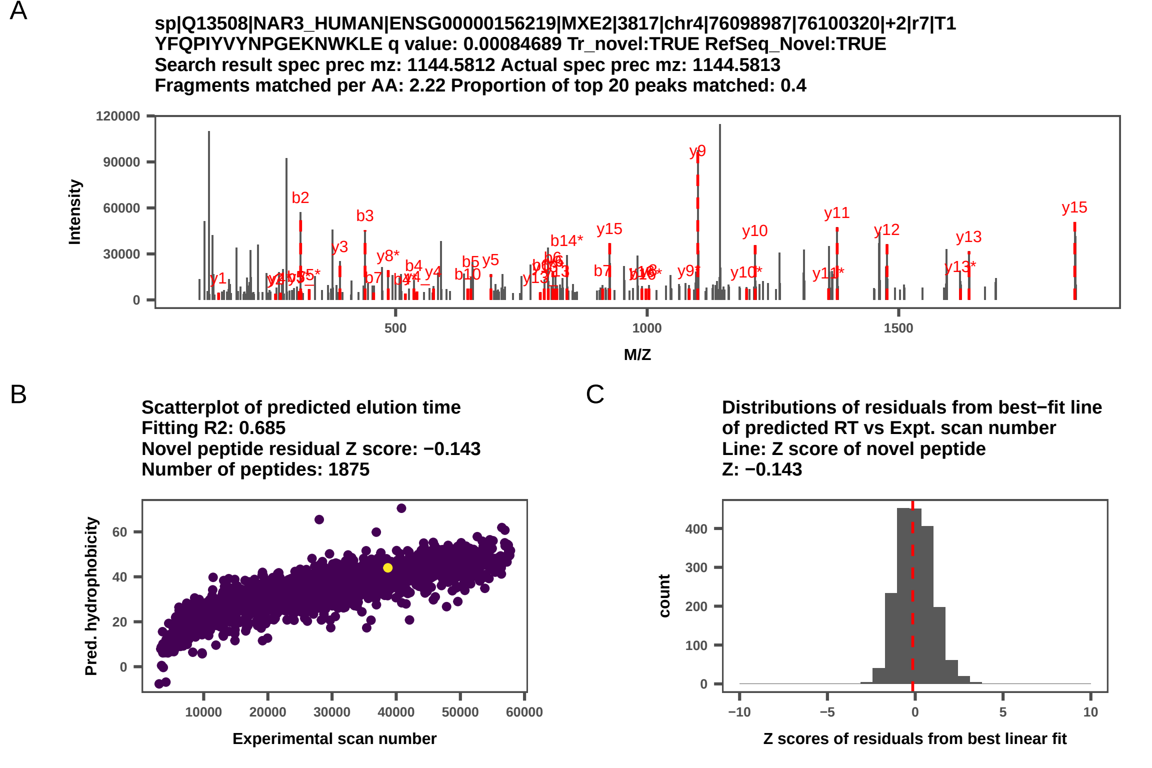
A
sp|Q13508|NAR3_HUMAN|ENSG00000156219|MXE2|3817|chr4|76098987|76100320|+2|r7|T1
YFQPIYVYNPGEKNWKLE q value: 0.00084689 Tr_novel:TRUE RefSeq_Novel:TRUE
Search result spec prec mz: 1144.5812 Actual spec prec mz: 1144.5813
Fragments matched per AA: 2.22 Proportion of top 20 peaks matched: 0.4
Intensity
120000
90000
60000
30000
0
500
1000
1500
M/Z
y9
b2
b3
y3
y8*
b4
y4
b5
y5
b10
b14*
y15
y10
y11
y12
y13
y13*
y15
y1
b7
y9*
y10*
B
Scatterplot of predicted elution time
Fitting R2: 0.685
Novel peptide residual Z score: −0.143
Number of peptides: 1875
Pred. hydrophobicity
60
40
20
0
10000
20000
30000
40000
50000
60000
Experimental scan number
C
Distributions of residuals from best−fit line
of predicted RT vs Expt. scan number
Line: Z score of novel peptide
Z: −0.143
count
400
300
200
100
0
−10
−5
0
5
10
Z scores of residuals from best linear fit
